# N-Carbamylglutamate Improves Reproductive Performance and Alters Fecal Microbiota and Serum Metabolites of Primiparous Sows during Gestation after Fixed-Time Artificial Insemination

**DOI:** 10.3390/biology11101432

**Published:** 2022-09-30

**Authors:** Tao Feng, Linli Xiao, Jiahua Bai, Hongxiang Ding, Liyan Pang, Yuqing Song, Yusheng Qin, Xiaoling Xu, Jing Wang, Yan Liu

**Affiliations:** 1 Institute of Animal Husbandry and Veterinary Medicine (IAHVM), Beijing Academy of Agriculture and Forestry Sciences (BAAFS), Beijing 100097, China; 2 Joint Laboratory of Animal Science between IAHVM of BAAFS and Division of Agricultural Science and Natural Resource of Oklahoma State University, Beijing 100097, China

**Keywords:** N-carbamylglutamate, fixed-time artificial insemination, sow reproductive performance, microbial community, metabolic profiles

## Abstract

**Simple Summary:**

Larger litter size (born alive) and higher uniformity of piglets’ birth weight are the most desired characteristics in pig production. Application of N-carbamylglutamate (NCG) was shown to increase litter size and decrease the coefficient of variation of piglets’ birth weight from sows after conventional artificial insemination. In this study, we report that, in sows after fixed-time artificial insemination (FTAI), NCG improved the number of piglets born alive and the coefficient of variation of birth weight through direct effects (increased serum NCG levels) and indirect effects (altered intestinal microbiome and serum metabolites). These findings indicate that NCG can improve reproductive performance by improving both the number of piglets born alive and the uniformity of piglets’ birth weight in sows after FTAI.

**Abstract:**

N-carbamylglutamate (NCG) supplementation during gestation improves reproductive performance in sows after conventional artificial insemination. However, whether NCG can improve reproductive performance and change fecal microbiota and serum metabolite levels during pregnancy in sows after fixed-time artificial insemination (FTAI) remains unclear. Two hundred multiparous sows were assigned a diet from mating until farrowing: control (corn–soybean meal) or NCG supplementation (0.05% NCG). At days 30, 70, and 110 of gestation and after farrowing, maternal microbial diversity and serum metabolites were studied. Supplementation of NCG increased the number of piglets born alive and the litter weight (all *p* < 0.05) and altered the fetal microbial community during gestation. Some genera were particularly abundant at different time points during gestation and after farrowing, but none were commonly abundant across all four time points. Metabolic analysis revealed that NCG supplementation significantly increased the serum concentrations of NCG, ferulic acid, cinnamoylglycine, 3-phenyllactic acid, and gamma-glutamylglutamic acid in the NCG group compared with levels in the control group. Our results reveal that NCG supplementation during gestation improves reproductive performance in sows after FTAI, exerting both direct (increased serum NCG levels) and indirect effects (altered intestinal microbiome and serum metabolites) on sow reproduction and, ultimately, improving placental and fetal development.

## 1. Introduction

Over the past few decades, many studies have explored the relationship between intestinal microorganisms and host metabolism. Intestinal microorganisms are involved in many host metabolic processes, including regulation of energy absorption and transportation, participation in lipid metabolism, modulation of hormone secretion, resistance to inflammation, and prevention of diseases, which are of great significance to maintaining host health and intestinal homeostasis [1,2,3]. Maternal intestinal microorganisms are closely related to fetal intestinal colonization, and the maternal microbial environment will affect the development of the fetus [4]. Intestinal microorganisms also affect female reproduction [5]. Compared with low prolificacy sows (litter size less than 7), high prolificacy sows (litter size more than 15) had lower fecal microbial richness during late pregnancy and higher fecal microbial diversity during early stage after farrowing [6].

Studies have shown that the intestinal microbiome is affected by genetics, diet, environment, and other factors, of which diet seems to be the main factor [7]. Arginine (Arg) is an essential amino acid with various physiological functions. Dietary Arg deficiency can lead to animal growth retardation, intestinal disorders, reproductive dysfunction, immune and neurodevelopmental damage, and even death [8,9]. Administration of Arg in diets can improve the implantation of mammalian embryos, increase the survival rate of embryos and litter size [10], and produce polyamines and nitric oxide, which play a crucial role in placental angiogenesis and growth [11]. However, owing to its short half-life, fast metabolism, and high cost in mammals, Arg supplementation has certain limitations [12]. N-carbamylglutamate (NCG) is an analog of endogenous N-acetylglutamic acid (an activator of Arg synthesis), which has been shown to promote neonatal growth by increasing the level of circulating Arg [13]. At the same time, NCG reduces intrauterine growth restriction [14], alleviates oxidative stress, and inhibits intestinal inflammation [15]. Therefore, NCG is a widely used alternative to Arg. Many studies have shown that NCG can regulate placental function by regulating angiogenesis [16] and endometrial protein composition [17] and inhibiting embryonic trophoblast differentiation [18], thereby promoting placental development in sows. Only one published paper showed that NCG supplementation changed cecal bacterial flora in rabbits [19]. Whether NCG supplementation alters fecal microbiome diversity and composition during pregnancy in sows remains unclear.

Fixed-time artificial insemination (FTAI) technology, which originated in Germany [20] and has been being developed in China in recent years [21,22], is characterized by intervention with exogenous reproductive hormones to synchronize ovulation in batches of gilts or sows. Such hormones can stimulate follicular development and induce ovulation in sows [22], but FTAI does not change the litter sizes of replacement gilts and multiparous sows [21,22]. Thus, FTAI technology can improve the breeding utilization rate and reduce the number of non-productive days in sows, to increase the productive efficiency of pig farm management. Being fed NCG can increase litter size in sows subjected to conventional artificial insemination [18], but whether NCG can increase litter size in sows subjected to FATI remains unknown.

Application of FTAI can increase ovulation in sows, but it has a limited effect on litter size, while NCG can increase litter size in sows subjected to conventional artificial insemination. However, few studies have investigated the effects of NCG on intestinal flora and serum metabolites in pregnant sows or any associations with reproductive performance, especially in sows after FTAI. This study aimed to investigate the effects of NCG supplementation on reproductive performance, intestinal microflora, and serum metabolites in sows subjected to FTAI.

## 2. Materials and Methods

### 2.1. Animals and Experimental Design

This experiment was conducted on a large pig farm with 3000 sows in Hebei Province. Two hundred Large White × Landrace crossbred sows from the same batch with normal estrus, uniform body condition score, good health, and parities of 3–5 were selected for breeding using FTAI technology. Twenty-four hours after weaning, the sows received 1000 IU equine chorionic gonadotropin (eCG; Ningbo Sansheng, Zhejiang, China), followed by 100 μg GnRH agonist (gonadorelin; Ningbo Sansheng, Zhejiang, China) after heat detection by boars, and were then mated using post-cervical artificial insemination twice (commercial mixed semen with three billion motile sperm in 80 mL, 8 h and 32 h after estrus) [22]. The sows were randomly divided into two groups: the control group was fed a basal diet, and the NCG group was fed a basal diet plus 0.05% NCG (commercial feed additive obtained from Animore Sci & Tech, Zhengzhou, Henan, China). Solid NCG pellets were added at 500 g/tonne and mixed using a mixer at the feed mill in the pig farm. The trial period was from sow mating to delivery. The pregnant sows were housed in individual stalls (2.50 × 0.85 m) during pregnancy, were maintained at 20–23 °C with 55–60% relative humidity, and were transferred to individual farrowing pens (2.50 × 2.70 m) one week before delivery. The housing conditions were maintained at 22–24 °C with 57–62% relative humidity around farrowing crates.

The basic diet of the sows was supplied by CP FEED (No. 566 produced in Inner Mongolia, China) with nutrient composition of crude protein ≥ 13.0%, crude ash ≤ 7.0%, crude fiber ≤ 8.0%, calcium ≥ 0.85%, phosphorus ≥ 0.4%, lysine ≥ 0.55%, and phytase ≥ 500 FTU/kg. Drinking water was provided ad libitum during the experiment. Feeding management, vaccinations, biosecurity, and disinfection procedures were carried out according to the conventional protocol in pig farms. The feeding behavior and health status of the sows were kept under continuous observation. Sows diagnosed with abortion or severe hoof disease were eliminated from the experiment. Food consumption during pregnancy and after delivery was gradually increased from 1.5–2.5 kg/d over days 1–85 of pregnancy, to reach 3.5 kg/d before delivery, and was further increased by 0.5 kg/d after delivery. 

### 2.2. Sampling and Measurement of Reproductive Performance

Eight sows were randomly selected from each group on the 30th day of pregnancy (D30). On D30, the 70th day of pregnancy (D70), the 110th day of pregnancy (D110), and after farrowing (F), fresh rectal feces (100 g) were collected from the same sows, quickly frozen in liquid nitrogen, and stored at −80 °C for microbial diversity analysis. Simultaneously, 10 mL blood samples from the same sows were collected from their ear veins before morning feeding and coagulated in coagulation tubes. The serum was separated by centrifugation at 3000 g for 20 min at 4 °C, transferred to sterilized centrifuge tubes, and stored at −20 °C for determination of metabolite levels.

On the day of delivery, the total piglets born and piglets born alive were recorded for all sows, in which the birth weight and placental weight were recorded for about 50 sows in each group, and the coefficients of variation of birth weight and placental weights were calculated. The difference between reproductive performances in the two groups was analyzed using Student’s t-test and is shown as mean ± standard deviation. A *p*-value less than 0.05 indicated statistical significance.

### 2.3. Fecal Microbial Diversity Analysis

Microbial genomic DNA was extracted from fecal samples using the E.Z.N.A. soil DNA Kit (Omega Bio-Tek, Norcross, GA, USA), in accordance with the instructions of the manufacturer. The DNA extract was checked on 1% agarose gel, and DNA concentration and purity were determined using a NanoDrop 2000 UV-vis spectrophotometer (Thermo Fisher Scientific, Waltham, MA, USA). The hypervariable region V3-V4 of the bacterial 16S rRNA gene was amplified with primer pairs 338F (5′-ACTCCTACGGGAGGCAGCAG-3′) and 806R (5′-GGACTACHVGGGTWTCTAAT-3′), using an ABI GeneAmp 9700 PCR thermocycler (Applied Biosystems, Foster City, CA, USA). PCR amplification of the 16S rRNA gene was performed as follows: initial denaturation at 95 °C for 3 min, followed by 27 cycles of denaturation at 95 °C for 30 s, annealing at 55 °C for 30 s, extension at 72 °C for 45 s, and a single extension at 72 °C for 10 min, ending at 4 °C.

The PCR product was purified and sequenced on an Illumina MiSeq PE 300 platform/NovaSeq PE 250 platform (Illumina, San Diego, CA, USA), in accordance with the standard protocls of Majorbio Bio-Pharm Technology Co. Ltd. (Shanghai, China). Operational taxonomic units (OTUs) with a 97% similarity cut-off were clustered using UPARSE version 7.1, and chimeric sequences were identified and removed. The taxonomy of each OTU representative sequence was analyzed by RDP Classifier version 2.2 against the 16S rRNA database (Silva v138) (https://www.arb-silva.de/documentation/release-138/, accessed on 16 September 2022), using a confidence threshold of 0.7 [23]. Alpha diversity (Shannon and Simpson estimators were used for diversity evaluation, and Chao and ACE estimators were used for abundance evaluation) was analyzed using Mothur v. 1.31.2. Principal coordinates analysis (PCoA) was used to visualize differences in fecal community composition, reflecting beta diversity. The linear discriminant analysis effect size (LefSe) algorithm was used to identify differences between taxa in different groups. The biomarkers of LefSe analysis conducted in the microbiota study had an effect-size threshold of 2.5. GraphPad Prism (v. 8.0.2; GraphPad Software, San Diego, CA, USA) was used to analyze the abundances of flora. The 64 raw reads were approved by the NCBI Sequence Read Archive (SRA) database (https://www.ncbi.nlm.nih.gov/bioproject/PRJNA870745, accessed on 16 September 2022), with accession number PRJNA870745.

### 2.4. Serum Metabolomics Analysis

Using a 100 μL serum sample in a 2 mL centrifuge tube, metabolites were extracted using a 400 µL methanol:water (4:1, *v*/*v*) solution, with 0.02 mg/mL L-2-chlorophenylalanin as the internal standard. The mixture was allowed to settle at −10 °C and treated by high-throughput tissue crusher Wonbio-96c (Shanghai Wanbo Biotechnology, Shanghai, China) at 50 Hz for 6 min, followed by ultrasound at 40 kHz for 30 min at 5 °C. The sample was kept at −20 °C for 30 min and centrifuged for 15 min (4 °C, 13,000× *g*). The supernatant was then transferred to a sample vial for LC-MS/MS analysis. The data were then imported into the metabolomics software Progenesis QI 2.3 (Waters Corporation, Milford, MA, USA) for preprocessing. Pooled quality control samples (QC) were used for system conditioning and quality control. MS/MS signals were matched with reliable biochemical databases Human Metabolome database (HMDB) (http://www.hmdb.ca/).

The preprocessed data were uploaded to the MSG BioCloud platform (https://cloud.majorbio.com) for data analysis. Principal component analysis (PCA) and orthogonal least squares discriminant analysis (OPLS-DA) were performed using the R ropls package (Version1.6.2). The selection of significantly different metabolites was based on the variable importance in projection (VIP) scores obtained by the OPLS-DA model and Student’s t-test. Metabolites with VIP > 1 and *p* < 0.05 were significantly different. Differential metabolites were screened, and related pathways were obtained through metabolic pathway annotation in the KEGG database (https://www.kegg.jp/kegg/pathway.html). The Python software package scipy.stats was used for pathway enrichment analysis, and the most relevant biological pathways were obtained using Fisher’s exact test.

### 2.5. Correlation between Serum Metabolites and Fecal Microbial Taxa

The correlation between relative abundance of fecal microbiota at genus levels and serum differential metabolites was analyzed by R package ggplot2. *p*-values less than 0.05 were considered to be significantly different.

## 3. Results

### 3.1. Sows and Litter Performance

Three sows were eliminated from the experiment, and 197 sows delivered successfully. The NCG supplementation from mating until delivery significantly increased the number of piglets born alive and fetal survival rates by 1.09 and 3.39%, respectively (*p* < 0.05; Table 1).

The litter birth weight of the NCG group was significantly higher than that of the control group (*p* < 0.05; Table 2); however, the average birth weight was not different (*p* > 0.05), but the coefficient of variation of birth weight decreased by 2.61% (*p* < 0.01).

### 3.2. Changes in Fecal Microorganisms

In the alpha diversity analysis, the Shannon index gradually decreased, and the Simpson, Ace, and Chao indices gradually increased during pregnancy (Table 3), indicating that species diversity gradually decreased, and richness steadily increased. However, NCG supplementation alleviated this decreasing trend in species diversity. No significant differences were detected between the Shannon, Simpson, ACE, or Chao indices in the control and NCG groups at D30 or D70 during pregnancy or after delivery (*p* > 0.05). At D110, there were significant differences between the Shannon and Simpson indices in the control and NCG groups (*p* < 0.05). These results indicated that administering NCG significantly increased microbial diversity in late pregnancy. In addition, the coverage index was greater than 99% for the control and NCG groups, indicating that the sequence in the sample was detected with high probability.

**Figure 1:**
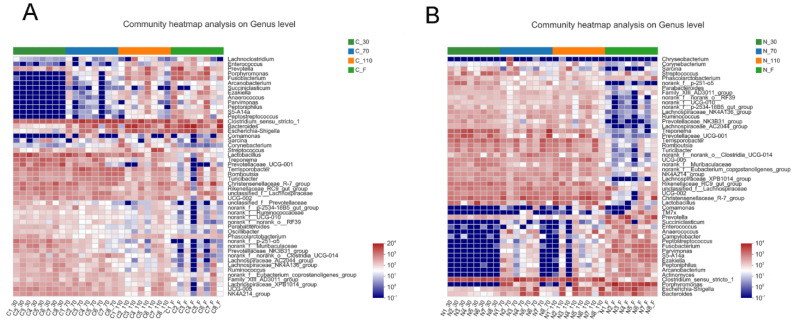
The dynamics in fecal microbiota development during pregnancy and after farrowing of sows in the control (**A**) and NCG (**B**) groups. NCG: N-carbamylglutamate; *n* = 8.

The top 50 genera, based on the microbial abundance in the control group (Figure 1A) and NCG group (Figure 1B), were shown through community heatmap. During pregnancy and after delivery, sows had dynamics in the fecal microbiota development trajectory. The overall composition of the fecal microbiota was altered at different stages after NCG supplementation.

**Figure 2:**
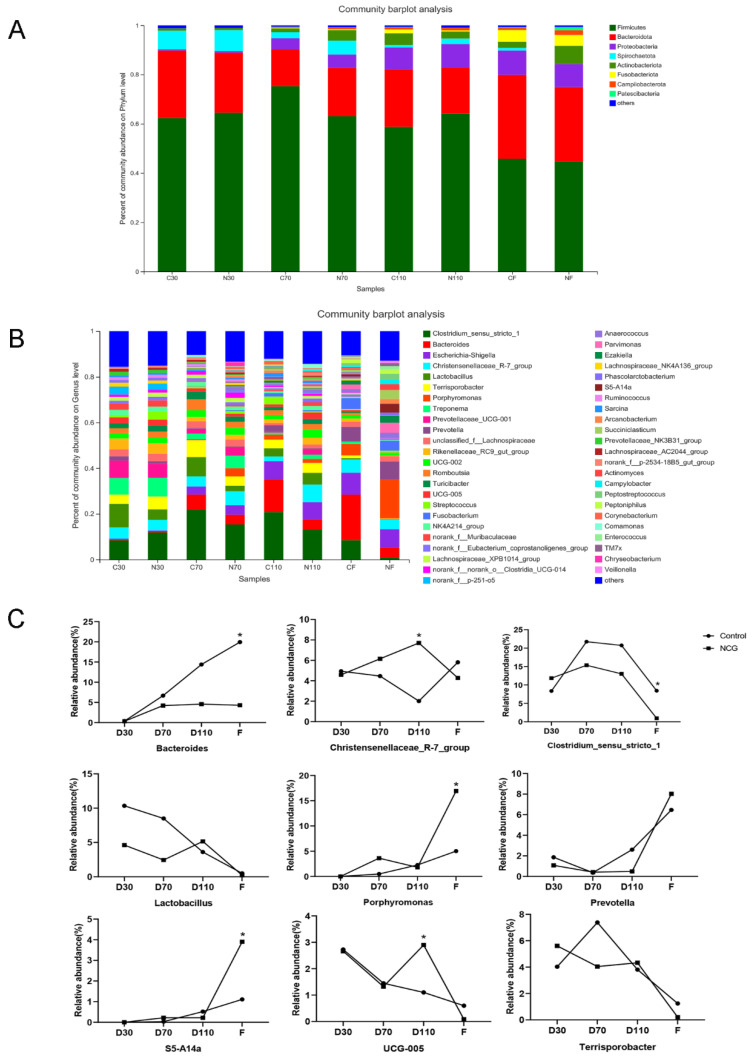
The distribution of fecal gut microbiota of sows during pregnancy and after farrowing in the control and NCG groups. (**A**) The distribution of bacterial phyla in fecal samples of sows during pregnancy and after farrowing in the control and NCG groups. (**B**) The distribution of bacterial genera in fecal samples of sows during pregnancy and after farrowing in the control and NCG groups. (**C**) Microbiota profiles showing relative abundance of certain bacterial genera in fecal samples of sows during pregnancy and after farrowing in the control and NCG groups. NCG: N-carbamylglutamate; *n* = 8; * denotes *p* < 0.05.

At the phylum level (Figure 2A), Firmicutes and Bacteroidetes were dominant in the control and NCG groups at different pregnancy stages and after farrowing. As pregnancy progressed, the abundance of Proteobacteria, Actinobacteria, and Fusobacteria gradually increased, while the abundance of Spirochetes gradually decreased. At the genus level (Figure 2B), as pregnancy progressed, the intestinal flora showed dynamic changes. Overall, the administration of NCG had little effect on intestinal microorganisms in early pregnancy but had a relatively significant impact later. As shown in Figure 2C, compared with that in the control group, the abundance of Clostridium_sensu_stricto_1 and Terrisporobacter increased, and the abundance of Lactobacillus and Prevotella decreased 30 days into pregnancy. In the middle of pregnancy, the abundance of Christensenellacea_R-7_group and Porphyromonas increased, whereas the abundance of Bacteroides, Clostridium_sensu_stricto_1, Lactobacillus, and Terrisporobacter decreased in the sows of the NCG group. In the late stages of pregnancy, the abundance of Christensenellacea_R-7_group, Lactobacillus, and UCG-005 increased, whereas the abundance of Bacteroides, Clostridium_sensu_stricto_1, and Prevotella decreased in the NCG group.

**Figure 3:**
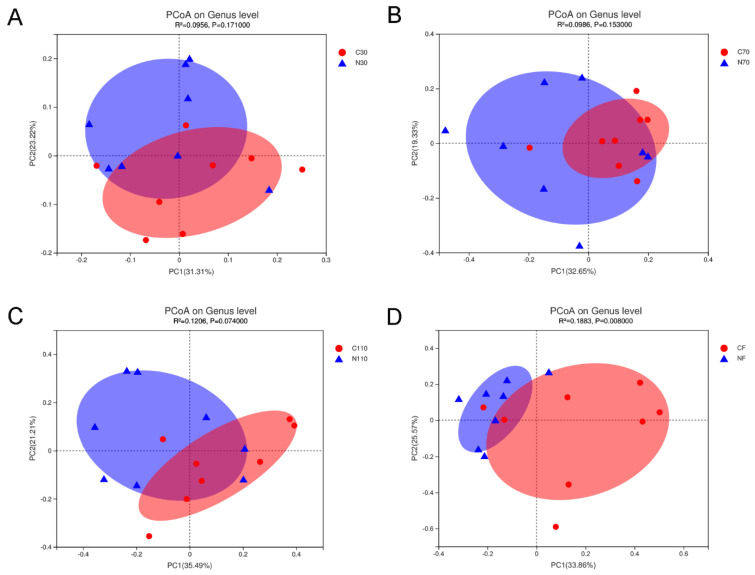
Score plots of PCoA of fecal microbiota in sows during pregnancy and after farrowing in the control and NCG groups at the genus level based on Bray–Curtis dissimilarity. (**A**) PCoA score plot at D30 of pregnancy. (**B**) PCoA score plot at D70. (**C**) PCoA score plot at D110. (**D**) PCoA score plot after farrowing. NCG: N-carbamylglutamate; C30, C70, C110, and CF represent control group at D30, D70, D110 of pregnancy, and after farrowing, respectively; N30, N70, N110, and NF represent NCG group at D30, D70, D110 of pregnancy, and after farrowing, respectively; *n* = 8.

Using PCoA analysis based on Bray–Curtis distance revealed that the fecal flora composition of the control and NCG groups at different pregnancy stages and after delivery differed by NCG supplementation (Figure 3). As the pregnancy progressed, the cluster of scatter plots in the two groups separated clearly. Additionally, R^2^ gradually increased, and *p*-value gradually decreased as gestational age increased, which also showed that the effects of NCG on intestinal flora gradually increased with the progress of gestation in sows subjected to FTAI.

**Figure 4:**
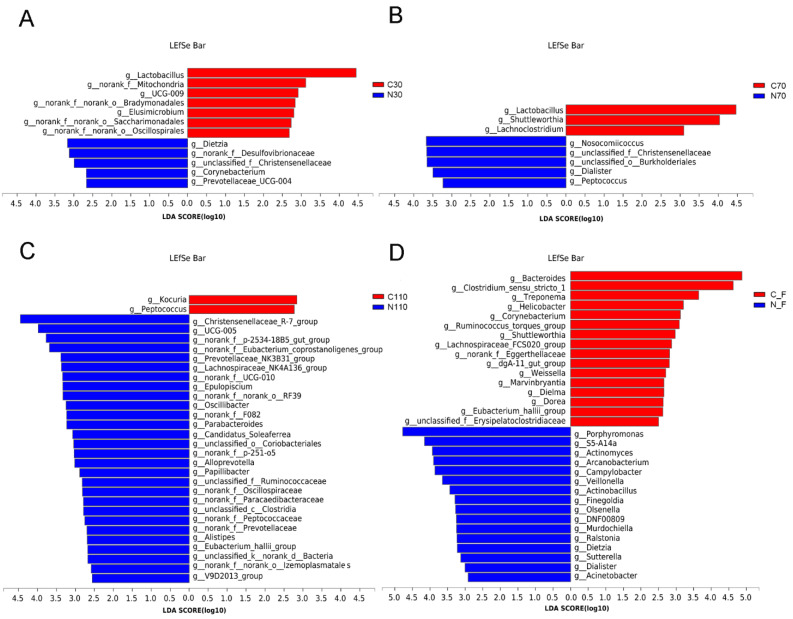
LEfSe analysis to identify the differentially abundant taxa genera (with LDA ≥ 2.5) of fecal microbiota of sows during pregnancy and after farrowing in the control and NCG groups. (**A**) The distribution of bacterial genera in fecal samples of sows at D30 of pregnancy. (**B**) The distribution of bacterial genera in fecal samples of sows at D70. (**C**) The distribution of bacterial genera in fecal samples of sows at D110. (**D**) The distribution of bacterial genera in fecal samples of sows after farrowing. NCG: N-carbamylglutamate; C30, C70, C110, and CF represent control group at D30, D70, D110 of pregnancy, and after farrowing, respectively; N30, N70, N110, and NF represent NCG group at D30, D70, D110 of pregnancy, and after farrowing, respectively; *n* = 8.

The LEfSe analysis was used to screen key microorganism differences in the control and NCG groups. Several bacterial genera were shown to be related to NCG supplementation based on linear discriminant analysis (LDA ≥ 2.5), and the number of microorganisms in the NCG group increased gradually, while the number of microorganisms in the control group decreased gradually during pregnancy, which further indicated that NCG had a significant impact on the microbial flora of sows during pregnancy (Figure 4). At D30, seven genera, including Lactobacillus and Elusimicrobium, were enriched in the control group, and five genera, including Dietzia and Corynebacterium, were enriched in the NCG group. At D70, Lactobacillus, Shuttleworthia, and Lachnoclostridium, were significantly enriched in the control group, whereas 28 genera, including Nosocomiicoccus and Dialister, were significantly enriched in the NCG group. At D110, Kocuria and Peptococcus were significantly enriched in the control group, and 28 genera, including Christensenellaceae_R-7_group and UCG-005, were significantly enriched in the NCG group. After delivery, 16 genera were significantly enriched in the control group, including Bacteroides and Clostridium_sensu_stricto_1, and 16 genera, including Porphyromonas and S5-A14a, were significantly enriched in the NCG group.

### 3.3. Changes in Serum Metabolites

**Figure 5:**
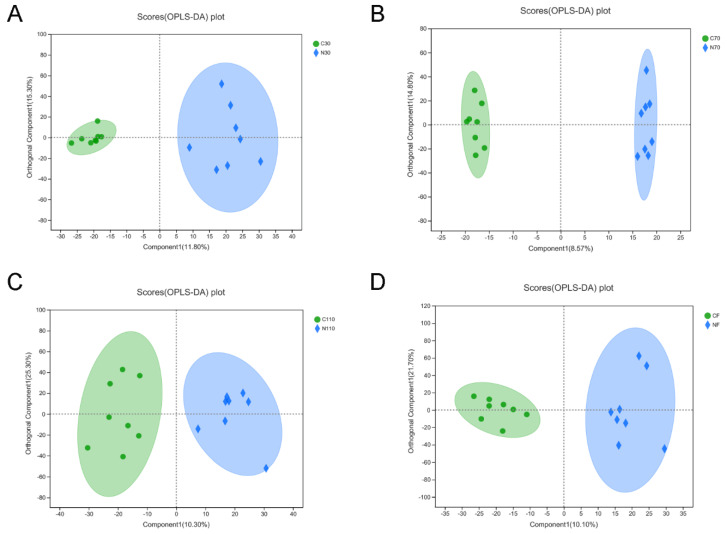
Score plots of OPLS-DA models of serum metabolite in fecal samples of sows during pregnancy and after farrowing in the control and NCG groups. (**A**) OPLS-DA score plot of serum metabolites in sows on D30 of pregnancy. (**B**) OPLS-DA score plot on D70. (**C**) OPLS-DA score plot on D110. (**D**) OPLS-DA score plot after farrowing. NCG: N-carbamylglutamate; C30, C70, C110, and CF represent control group at D30, D70, D110 of pregnancy, and after farrowing, respectively; N30, N70, N110, and NF represent NCG group at D30, D70, D110 of pregnancy, and after farrowing, respectively; *n* = 8.

Results from OPLS-DA showed that 64 differential metabolites were screened at D30, of which 16 were significantly upregulated and 48 were significantly downregulated (Figure 5). A total of 45 differential metabolites were screened at D70, of which 12 were upregulated and 33 were downregulated. A total of 39 differential metabolites were screened at D110, of which 23 were upregulated and 16 were downregulated. In total, 84 differential metabolites were screened after childbirth, of which 42 were upregulated and 42 were downregulated. Details of the differential metabolites at the four time points after NCG administration are shown in the Table S1.

**Figure 6:**
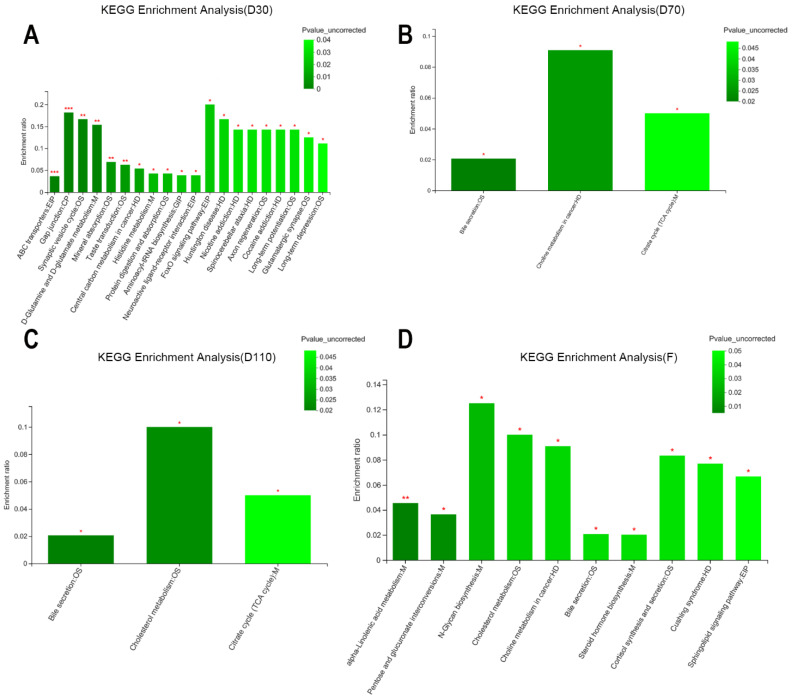
The identified functional KEGG pathway annotation of differential serum metabolites in sows during pregnancy and after farrowing in the control and NCG groups. (**A**) KEGG pathway annotation of serum metabolites in sows on D30 of pregnancy. (**B**) KEGG pathway annotation of serum metabolites on D70. (**C**) KEGG pathway annotation of serum metabolites on D110. (**D**) KEGG pathway annotation of serum metabolites after farrowing. D30: the 30th day of pregnancy; D70: the 70th day of pregnancy; D110: the 110th day of pregnancy; F: after farrowing; ***: the KEGG pathway was significantly changed in NCG group compared with control group (*p* < 0.001); **: *p* < 0.01; *: *p* < 0.05; *n* = 8.

The differential metabolites altered by NCG supplementation were annotated in the KEGG pathway analysis. At D30, the 20 annotated KEGG pathways included ABC transporters, gap junction, and D-glutamine and D-glutamate metabolism. As pregnancy progressed, the number and types of KEGG pathways tended to be consistent at D70 and D110, which were mainly bile secretion and citrate cycle (TCA cycle). After delivery, 10 KEGG pathways were annotated, including α-linolenic acid metabolism, pentose and glucuronate interconversions, and N-glycan biosynthesis (Figure 6). The differential metabolites were classified and analyzed using Human Metabolome Database compounds at the classification level of “super class”. Among all blood metabolites, the proportion of lipids and lipid-like molecules was highest in this study, followed by organic acids and derivatives and organic oxygen compounds (data not shown).

**Figure 7:**
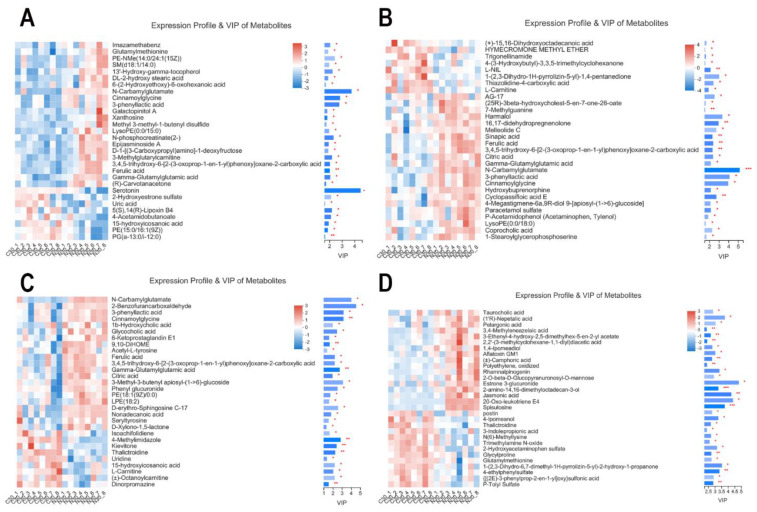
Variable importance in projection (VIP) scores of serum metabolites in sows during pregnancy and after farrowing in the control and NCG groups. (**A**) VIP scores of serum metabolites in sows on D30 of pregnancy. (**B**) VIP scores on D70. (**C**) VIP on D110. (**D**) VIP scores after farrowing. C30, C70, C110, and CF represent control group at D30 of pregnancy, D70 of pregnancy, D110 of pregnancy, and after farrowing, respectively; N30, N70, N110, and NF represent NCG group at D30 of pregnancy, D70 of pregnancy, D110 of pregnancy, and after farrowing, respectively; Red represents a higher expression abundance of metabolite, while blue represents lower; ***: the expression abundance of metabolite was significantly different between NCG group and control group (*p* < 0.001); **: *p* < 0.01; *: *p* < 0.05; *n* = 8.

According to the weighted coefficient of the OPLS-DA model, VIP was used to rank the differential contributions of metabolites in the control and NCG groups. At D30, according to the VIPs of the metabolites, the top four were serotonin > N-carbamylglutamate > cinnamoylglycine > 3-phenyllactic acid (Figure 7A). At D70, the top four were N-carbamylglutamate > 3-phenyllactic acid > cinnamoylglycine > harmalol (Figure 7B). At D110, the top four were 2-benzofurancarboxaldehyde > N-carbamylglutamate > 3-phenyllactic acid > and cinnamoylglycine (Figure 7C). After delivery, the top four were estrone 3-glucuronide > 20-oxo-leukotriene E4 > jasmonic acid > (1’R)-nepetalic acid (Figure 7D). The metabolites differentially expressed during pregnancy in the NCG group were N-carbamylglutamate, ferulic acid, cinnamoylglycine, 3-phenyllactic acid, and gamma-glutamylglutamic acid.

### 3.4. Correlation between Serum Metabolites and Fecal Microbial Taxa

**Figure 8:**
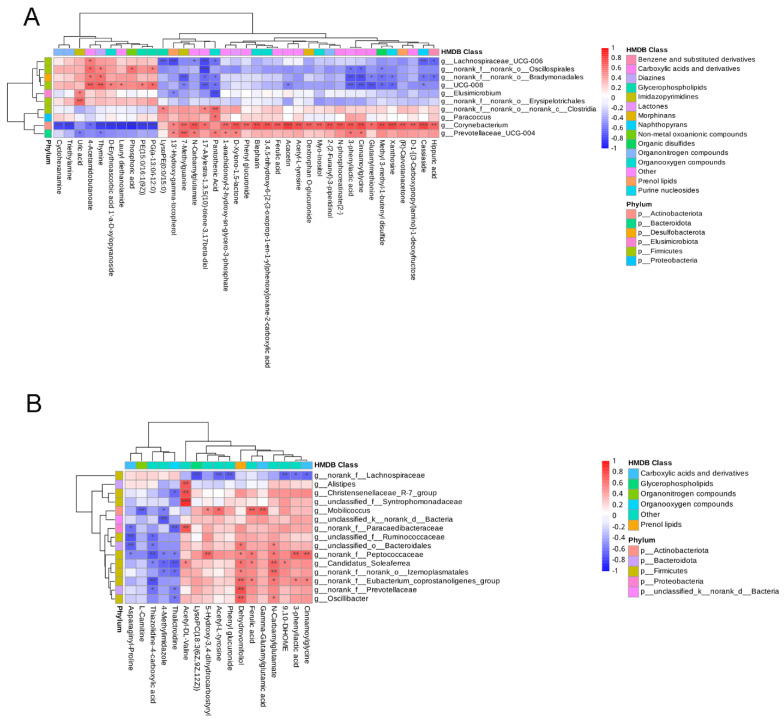
Heatmap showing the correlation between fecal microorganism and serum metabolome in sows at different pregnancy stages. (**A**) Pearson correlation coefficients in sows on D30 of pregnancy. (**B**) Pearson correlation coefficients on D110 of pregnancy. Red represents a positive correlation, while blue represents a negative correlation; ***: significant correlation between the serum metabolites and the fecal microbes (*p* < 0.001); **: *p* < 0.01; *: *p* < 0.05; *n* = 8.

In order to identify the relevance between serum metabolites (VIP > 1, *p* < 0.05) and fecal microbes (genus level), a correlation matrix was conducted based on the Pearson’s correlation coefficient (Figure 8). The relevance between serum metabolites and fecal microbes seems complicated and irregular during gestation and after farrowing. Concerning the metabolites differentially expressed during pregnancy in the NCG group, N-carbamylglutamate was positively associated with Corynebacterium and Prevotellaceae_UCG-004, while it was negatively associated with Lachnospiraceae_UCG-006 at D30 (Figure 8A). At D110, N-carbamylglutamate was positively connected with Candidatus_Soleaferrea and Oscillibacter, but none of the genera had negatively connected with it (Figure 8B). Ferulic acid was positively associated with Corynebacterium at D30, while it was positively associated with Mobilicoccus and Candidatus_Soleaferrea at D110 (Figure 8A,B).

## 4. Discussion

In large-scale pig farming, the number of piglets born alive, birth weight, and uniformity of piglets are important indicators of the reproductive efficiency and economic benefits of sows. Many studies have shown that NCG supplementation during pregnancy can improve the birth weight of piglets, reduce the coefficient of variation of birth weight, and improve pregnancy outcomes in sows [18,24,25,26]. However, no study has evaluated the effects of NCG administration on reproductive performance in sows after FTAI. In this study, supplementation of the basal diet with 0.05% NCG during pregnancy significantly improved the number of piglets born alive to sows subjected to FTAI (*p* < 0.05), which is consistent with the increase in piglets born alive to sows subjected to conventional artificial insemination and fed diets supplemented with NCG [18,24,25,26]. Consequently, administration of NCG during FTAI pregnancy significantly increased litter weight (*p* < 0.05). However, NCG did not significantly increase average placental weight (*p* > 0.05). In addition, NCG supplementation reduced the coefficients of variation of birth but not the placental weights of piglets after FTAI, which is consistent with data for sows subjected to conventional artificial insemination supplemented with NCG [26]. Supplementation of NCG can increase concentrations of Arg-family amino acids, which may activate the PI3K/Akt/mTOR signaling pathway during the peri-implantation period, thereby increasing litter size [13]. Our previous studies have also shown that NCG regulates embryonic function by increasing proliferation and inhibiting differentiation of pig placental trophoblast cells [18]. These results show that administering NCG improves reproductive efficiency by increasing fetal survival rate and litter weight in sows subjected to FTAI.

Intestinal microbiota plays an essential role in the health, physiology, and metabolic activities of the host. Changing the abundance of a specific intestinal microorganism may affect some metabolic functions and even whole-body mechanisms. In this study, with the progress of pregnancy, the species and abundance of fecal microorganisms in sows significantly changed, which is consistent with previous studies [27]. Alteration of alpha diversity in sow fecal bacteria showed that microbial diversity decreased and richness increased over days 30–110 of pregnancy. Kong et al. [28] and Koren et al. [29] found that microbial community diversity and richness were lower at the end of pregnancy than earlier, of which the alteration of richness is contrary to the results in the present study. Whether NCG supplementation alters fecal microbiome diversity and composition during pregnancy in sows subjected to FTAI remains unclear. In the present study, gestational NCG supplementation influenced the fecal microbial community structure of pregnant sows subjected to FTAI to a certain extent. No significant differences were identified between the alpha diversities of fecal microorganisms at D30, D70, or after farrowing in the NCG and control groups. Specifically, the Shannon index was higher, whereas the Simpson index was lower in the NCG group than in the control group on D110 (*p* < 0.05), indicating that NCG supplementation significantly increased fecal microbial diversity during late pregnancy. However, the results of the beta diversity analysis showed that there was a separation between the fecal microorganisms in the NCG and control groups. Interestingly, as gestational age increased, the distance between sample clusters in the NCG and control groups gradually increased, indicating that the effect of NCG on intestinal flora was enhanced as pregnancy progressed. To the best of our knowledge, this is the first study to demonstrate that NCG modulates the microbial community structure in pregnant sows.

Intestinal microorganisms are closely associated with energy metabolism, hormone levels, immune regulation, and inflammatory response. Firmicutes and Bacteroidetes are two dominant bacteria in the intestines of sows and are critical indicators of body health [30,31]. Similar to previous studies, the present study showed that Firmicutes and Bacteroidetes were the two most predominant phyla, followed by Proteobacteria and Spirochetes, in pregnant sows. The abundance of Spirochetes gradually decreased with gestation progress, and NCG supplementation seemed to alleviate this decrease. In contrast, the abundance of Proteobacteria gradually decreased during pregnancy, but no clear change in abundance was found between the NCG and control groups. All of the above results further verified that the composition of the core gut microbiota was essentially stable during pregnancy [32], while some components were dynamically affected by environmental stresses [33] or nutrient supplementation [34]. 

Although no statistical differences were detected, NCG increased the Firmicutes to Bacteroidetes ratio by 16% and 36% at D30 and D110, respectively. The increased ratio may prompt energy extraction from food to support the energy supply [35]. The period from D20 to D70 is for placental growth in preparation of rapid fetal growth between D70 and birth [36]. This finding may partially explain why sows in the NCG group had relatively higher placental and birth weights in the present study. Additionally, studies have shown that NCG plays an essential role in alleviating inflammation and enhancing adaptability by regulating intestinal microbes [19]. Inflammation is one of the main reasons for low reproductive performance in sows [37]. As pregnancy progresses, the abundance of inflammatory-related flora, mainly Proteobacteria, Actinobacteria, and Fusobacteria, increase in the intestinal microbiome [3,38], especially in late pregnancy [29]. In our study, NCG reduced the abundance of Actinobacteria and Fusobacteria but had little effect on the abundance of Proteobacteria at D110, indicating that NCG might alleviate the occurrence of inflammatory responses during late pregnancy and indirectly increase reproductive performance. In addition, several genera were identified by LEfSe analysis at different time points during gestation and after farrowing, but none of them were detected among the samples as common at all four time points. The number of bacterial genera significantly associated with NCG supplementation gradually increased during pregnancy, indicating that NCG continued to enhance the alteration of the microbial flora of the sows throughout pregnancy.

Due to the significant differences in fecal microbiota, alterations in serum metabolites after NCG supplementation were also investigated during gestation. Serum biochemical parameters are usually used as biomarkers to indicate body fitness and physiological conditions [39]. Non-targeted LC-MS metabolomics technology was used to describe the serum metabolite profiles in the NCG and control groups at different pregnancy stages. Several differential metabolites were screened based on *p* and VIP values and were annotated into specific KEGG pathways. At D30, 64 differential metabolites were annotated as ABC transporters, gap junctions, and D-glutamine and D-glutamate metabolism, among others. The transmembrane ATP-binding cassette (ABC) transporter regulates steroid synthesis, fertilization, embryo implantation, nutrient transport, and the immune response to participate in embryo implantation and placental development in early pregnancy, directly or indirectly [40,41]. With gestational progression, both differential metabolites (45 at D70 and 39 at D110) and their annotated KEGG pathways decreased (three KEGG pathways), and the KEGG pathways common to these two stages included bile secretion and citrate cycle. In late pregnancy, bile acid metabolism plays a vital role in regulating metabolic homeostasis, immune response, and antioxidation [42,43], while the citrate cycle is associated with energy metabolism [44]. Alterations in serum metabolites caused by NCG supplementation further confirmed that NCG plays an important role in embryo implantation, placenta formation, and fetal development in early pregnancy as well as in fetal and placental development in middle and late pregnancy. 

Interestingly, five serum metabolites were significantly upregulated in the NCG group compared to their levels in the control group, including NCG, ferulic acid, cinnamoylglycine, 3-phenyllactic acid, and gamma-glutamylglutamic acid. NCG supplementation increased serum NCG levels during gestation, indicating that NCG may be absorbed directly in addition to modulating the gut microbiota. NCG is an antioxidant that helps capture and neutralize free radicals, thereby preventing them from damaging the body. Previous studies have shown that NCG supplementation enhances the antioxidant capacity of the liver, spleen, and plasma of rats suffering from oxidative stress [45,46]; it also improves the oxidative stress state of the endometrium, upregulates the expression of endometrial receptivity-related proteins, and increases the survival rate of embryos during early pregnancy in sows [17,47]. Moreover, NCG promotes the synthesis of Arg in mammals and promotes the formation of nitric oxide and polyamines, which play essential roles in embryo implantation, embryo formation, fetal growth, and survival [13,24,25]. In this study, NCG increased the serum NCG level and the levels of other antioxidant metabolites (ferulic acid and citric acid) in sows subjected to FTAI. Ferulic acid is a phenolic antioxidant extracted from plants, mainly from whole grains, spinach, grapes, rhubarb, and cereal seeds, which has been shown to have a variety of physiological functions, including antioxidant [48], anti-inflammatory [49], anticancer [50], and vasodilatory activities [51]. During gestation, ferulic acid has been reported to alleviate preeclampsia [52] and gestational diabetes mellitus by relieving oxidative stress and reducing the inflammatory response [53]. Finally, 3-Phenyllactic acid is an organic acid widely found in honey and foods fermented by lactic acid bacteria. It is mainly produced by lactic acid bacteria through the degradation of phenylalanine, has more vigorous antibacterial activity than other organic acids, and is an ideal antibacterial compound [54,55]. Thus, in addition to its role in enhancing antioxidant activity, NCG seems to promote antibacterial activity in pregnant sows, something that should be validated in further studies. 

Several studies have confirmed that there is a close association between the metabolites and gut microbiota in sows [27,56]. Supplementation with NCG in male weanling Japanese White rabbits could promote the growth and immunity, increase nitrogen metabolism, and relieve inflammation of rabbits by regulating the intestinal microbial community [19]. Administration of NCG improved nitrogen utilization by increasing plasma amino acids, total essential amino acid, and total nonessential amino acid in fattening Holstein bulls [57]. In the present study, correlation analysis was performed to explore the associations between alterations of serum metabolites and fecal microbiota in sows administrated with NCG. Our analysis revealed that a number of metabolites in serum were positively or negatively correlated with the bacterial taxa in feces. These correlations might be important for NCG’s effects on sow reproduction. Moreover, the relevance between serum metabolites and fecal microbes identified during gestation and after farrowing seems complicated and irregularly, indicating that NCG might influence different microbiota and metabolites at different phases of gestation. Further studies are required to elucidate the effect of NCG on the relevance between serum metabolites and fecal microbes in sows’ reproduction. 

## 5. Conclusions

It can be concluded from the present study that NCG supplementation during gestation improves reproductive performance in sows subjected to FTAI. This improvement is associated with direct (increased serum NCG levels) and indirect effects (alterations in intestinal microbial composition and serum metabolites).

## Figures and Tables

**Table 1:** Effects of NCG on litter size in sows after FTAI.

Items	Control	NCG	*p*-Value
Sows	85	112	
Parity	3.60	3.86	
Total born	12.15 ± 2.32	12.88 ± 2.24	0.060
Born alive	10.76 ± 2.25	11.85 ± 2.26	0.004
Fetal survival rate (%)	88.59 ± 7.54 *	91.98 ± 5.71	<0.001

NCG: N-carbamylglutamate. *: showed that values within the same index have significant differences (*p* < 0.05). Data are expressed as mean ± standard deviation.

**Table 2:** Effects of NCG on litter weight and placental weight of sows after FTAI.

Items	Control	NCG	*p*-Value
Litters	47	49	
Litter birth weight (kg)	17.76 ± 4.61	19.49 ± 2.83	0.040
Average birth weight (kg)	1.49 ± 0.22	1.51 ± 0.22	0.840
Average placental weight (g)	274.83 ± 38.57	288.34 ± 5 1.74	0.480
Coefficient of variation of birth weight (%)	19.46 ± 4.46	16.85 ± 3.63	0.006
Coefficient of variation of placental weight (%)	22.38 ± 8.03	21.92 ± 5.58	0.370

NCG: N-carbamylglutamate. Coefficient of variation of birth weights of piglets based on the number of piglets born alive. Data are expressed as mean ± standard deviation.

**Table 3:** Effect of NCG on alpha diversity in sow feces.

Items		Shannon Index	Simpson Index	Ace Index	Chao Index	Coverage Index
D30	Control	3.50 ± 0.15	0.06 ± 0.02	185.65 ± 7.39	188.54 ± 10.55	0.9993 ± 0.0001
	NCG	3.51 ± 0.14	0.05 ± 0.01	187.46 ± 14.15	199.46 ± 23.17	0.9992 ± 0.0002
D70	Control	3.14 ± 0.27	0.10 ± 0.02	215.12 ± 41.40	224.51 ± 48.03	0.9990 ± 0.0003
	NCG	3.28 ± 0.36	0.09 ± 0.04	224.57 ± 38.88	230.04 ± 42.00	0.9990 ± 0.0002
D110	Control	3.11 ± 0.35 *	0.11 ± 0.04 *	213.18 ± 22.99	217.64 ± 23.46	0.9990 ± 0.0001
	NCG	3.38 ± 0.27 *	0.07 ± 0.03 *	230.27 ± 30.79	234.53 ± 31.90	0.9990 ± 0.0003
F	Control	2.70 ± 0.68	0.15 ± 0.10	155.60 ± 44.84	153.33 ± 50.18	0.9992 ± 0.0002
	NCG	3.03 ± 0.27	0.09 ± 0.03	169.59 ± 44.24	164.15 ± 38.24	0.9991 ± 0.0003

NCG: N-carbamylglutamate. Data are expressed as mean ± standard deviation. *: showed that values within the same index have significant differences (*p* < 0.05). D30: the 30th day of pregnancy; D70: the 70th day of pregnancy; D110: the 110th day of pregnancy; F: after delivery.

## Data Availability

All data that support the findings of this study are presented in the manuscript and supplementary materials of this article. Data sharing is not applicable to this article.

## Supplementary Materials

The following supporting information can be downloaded at: https://www.mdpi.com/article/10.3390/biology11101432/s1. Table S1: Details of the differential metabolites at the four time points after NCG administration.
