# Supplementary material for: N-Carbamylglutamate Improves Reproductive Performance and Alters Fecal Microbiota and Serum Metabolites of Primiparous Sows during Gestation after Fixed-Time Artificial Insemination
Source: Biology (Basel). 2022 Sep 30;11(10):1432. doi: 10.3390/biology11101432 (PMC9598523; doi:10.3390/biology11101432)
Supplement: Supplementary file 1 [file biology-11-01432-s001.zip › biology-1898596-supplementary.pdf]

Table S1. Details of the differential metabolites at the four time points after NCG administration

Group	Differential metabolites	VIP-Value	P-Value	Change trend
	D-Erythroascorbic acid 1'-a-D-xylopyranoside	1.06	0.024	↑
	5(S),14(R)-Lipoxin B4	2.20	0.014	↑
	PE-NMe(16:1(9Z)/14:1(9Z))	1.43	0.031	↑
	Triethylamine	1.29	0.005	↑
	Cyclohexanamine	1.08	0.031	↑
	PE(15:0/16:1(9Z))	1.88	0.010	↑
	PG(a-13:0/i-12:0)	1.66	0.007	↑
	15-hydroxyicosanoic acid	1.94	0.014	↑
	Phosphoric acid	1.07	0.007	↑
	Lauryl diethanolamide	1.02	0.024	↑
	L-Isoleucine	1.39	0.031	↑
	Thymine	1.56	0.031	↑
	4-Acetamidobutanoate	2.12	0.018	↑
	Serotonin	4.49	0.010	↑
	Uric acid	1.65	0.014	↑
	2-Hydroxyestrone sulfate	2.13	0.041	↑
	Hippuric acid	1.13	0.041	↓
	Pantothenic Acid	1.43	0.004	↓
	7-Methylguanine	1.38	0.010	↓
**D30**	Galactopinitol A	1.83	0.024	↓
	Methyl 3-methyl-1-butenyl disulfide	1.77	0.031	↓
	4-(Trimethylammonio)but-2-enoate	1.13	0.007	↓
	2-(2-Furanyl)-3-piperidinol	1.21	0.031	↓
	2,3-dihydroxy-1-(4-hydroxy-3-methoxyphenyl)-propan-1-one	1.24	0.041	↓
	Daidzein	1.46	0.041	↓
	PS(14:0/14:0)	1.36	0.005	↓
	17-HYDROXYPROGESTERONE	1.27	0.005	↓
	LysoPE(0:0/15:0)	1.98	0.031	↓
	17-Allylestra-1,3,5(10)-triene-3,17beta-diol	1.54	0.002	↓
	3-phenyllactic acid	2.79	0.018	↓
	Brevetoxin B4a	1.19	0.002	↓
	Glutamylmethionine	1.90	0.041	↓
	3-Methylglutarylcarnitine	2.21	0.018	↓
	D-Xylono-1,5-lactone	1.21	0.004	↓
	Gamma-Glutamylglutamic acid	2.06	0.018	↓
	Blepharin	1.46	0.004	↓
	Cassiaside	1.58	0.031	↓
	N-phosphocreatinate(2-)	2.33	0.018	↓

	Epijasminoside A	1.94	0.018	↓
	6-(2-Hydroxyethoxy)-6-oxohexanoic acid	1.72	0.031	↓
	Ferulic acid	2.02	0.007	↓
	Phenyl glucuronide	1.44	0.018	↓
	Acacetin	1.37	0.014	↓
	Imazamethabenz	2.05	0.024	↓
	LysoPE(0:0/22:1(13Z))	1.46	0.031	↓
	DL-2-hydroxy stearic acid	1.90	0.024	↓
	Dextrorphan O-glucuronide	1.63	0.005	↓
	1-arachidonoyl-2-hydroxy-sn-glycero-3-phosphate	1.58	0.024	↓
	PE-NMe(14:0/24:1(15Z))	2.43	0.041	↓
	PE-NMe(20:0/22:5(4Z,7Z,10Z,13Z,16Z))	1.29	0.041	↓
	PC(20:4(5Z,8Z,11Z,14Z)/P-18:0)	1.63	0.031	↓
	SM(d18:1/14:0)	1.98	0.041	↓
	13'-Hydroxy-gamma-tocopherol	2.25	0.024	↓
	LysoPC(P-18:0)	1.03	0.041	↓
	Cinnamoylglycine	2.85	0.014	↓
	N-formylanthranilic acid	1.12	0.031	↓
	(R)-Carvotanacetone	1.72	0.014	↓
	3,4,5-trihydroxy-6-[2-(3-oxoprop-1-en-1-yl)phenoxy]oxane-2-carboxylic acid	2.04	0.014	↓
	Acetyl-L-tyrosine	1.21	0.041	↓
	D-1-[(3-Carboxypropyl)amino]-1-deoxyfructose	1.91	0.031	↓
	Xanthosine	1.83	0.010	↓
	N-Carbamylglutamate	3.77	0.014	↓
	Myo-Inositol	1.06	0.031	↓
	L-Glutamate	1.31	0.041	↓
**D70**	L-NIL	2.12	0.007	↑
	Thiazolidine-4-carboxylic acid	2.43	0.018	↑
	DL-Norleucine	1.53	0.031	↑
	Theanine	1.13	0.031	↑
	HYMECROMONE METHYL ETHER	1.58	0.041	↑
	1-(2,3-Dihydro-1H-pyrrolizin-5-yl)-1,4-pentanedione	3.02	0.024	↑
	Triphenyl phosphate	1.12	0.031	↑
	4-(3-Hydroxybutyl)-3,3,5-trimethylcyclohexanone	1.59	0.031	↑
	(+)-15,16-Dihydroxyoctadecanoic acid	1.70	0.031	↑
	METHIONAL	1.51	0.031	↑
	L-Carnitine	1.93	0.010	↑
	Trigonellinamide	1.65	0.014	↑
	Pantothenic Acid	1.35	0.041	↓

	3,6,7-Trihydroxy-4'-methoxyflavone 7-rhamnoside	1.07	0.005	↓
	2-Pyrrolidinone	1.19	0.007	↓
	7-Methylguanine	1.55	0.005	↓
	Antiarol	1.55	0.014	↓
	4-Megastigmene-6a,9R-diol 9-[apiosyl-(1->6)-glucoside]	2.11	0.024	↓
	Cyclopassifloic acid E	2.74	0.007	↓
	PC(18:1(11Z)/18:3(9Z,12Z,15Z))	1.48	0.031	↓
	16,17-didehydropregnenolone	2.97	0.007	↓
	3-phenyllactic acid	4.11	0.014	↓
	P-Acetamidophenol (Acetaminophen, Tylenol)	1.88	0.024	↓
	N-Acetyl-DL-Glutamic acid	1.53	0.041	↓
	Citric acid	2.39	0.014	↓
	Gamma-Glutamylglutamic acid	2.15	0.024	↓
	LysoPC(20:3(5Z,8Z,11Z))	1.23	0.031	↓
	Coprocholic acid	2.29	0.041	↓
	D-1,5-Anhydrofructose	1.41	0.031	↓
	Ferulic acid	2.41	0.007	↓
	Melleolide C	2.73	0.041	↓
	AG-17	2.54	0.024	↓
	(25R)-3beta-hydroxycholest-5-en-7-one-26-oate	1.62	0.024	↓
	1-Stearoylglycerophosphoserine	1.70	0.041	↓
	Estrone glucuronide	1.21	0.041	↓
	LysoPE(0:0/18:0)	1.64	0.031	↓
	Hydroxybuprenorphine	2.15	0.010	↓
	1-Oleoylglycerophosphoserine	1.27	0.041	↓
	LysoPE(0:0/22:6(4Z,7Z,10Z,13Z,16Z,19Z))	1.55	0.041	↓
	Harmalol	3.36	0.018	↓
	Cinnamoylglycine	3.97	0.014	↓
	Paracetamol sulfate	2.16	0.010	↓
	Sinapic acid	2.32	0.005	↓
	3,4,5-trihydroxy-6-[2-(3-oxoprop-1-en-1-yl)phenoxy]oxane-2-carboxylic acid	2.34	0.007	↓
	N-Carbamylglutamate	5.17	0.001	↓
**D110**	2-Benzofurancarboxaldehyde	4.56	0.014	↑
	3-Methyl-3-butenyl apiosyl-(1->6)-glucoside	3.12	0.024	↑
	D-erythro-Sphingosine C-17	2.58	0.031	↑
	Nonadecanoic acid	1.82	0.014	↑
	3-phenyllactic acid	3.18	0.010	↑
	Seryltyrosine	1.55	0.031	↑
	D-Xylono-1,5-lactone	1.32	0.018	↑

	Citric acid	1.97	0.010	↑
	Monomenthyl succinate	1.23	0.024	↑
	Gamma-Glutamylglutamic acid	2.75	0.003	↑
	Ferulic acid	2.22	0.014	↑
	Phenyl glucuronide	2.52	0.018	↑
	9,10-DiHOME	1.66	0.005	↑
	LPE(18:2)	1.60	0.031	↑
	PE(18:1(9Z)/0:0)	1.73	0.018	↑
	1b-Hydroxycholic acid	2.00	0.041	↑
	Glycocholic acid	2.46	0.014	↑
	6-Ketoprostaglandin E1	1.61	0.014	↑
	3-Methyl-5-propyl-2-cyclohexen-1-one	1.21	0.014	↑
	Cinnamoylglycine	3.17	0.007	↑
	3,4,5-trihydroxy-6-[2-(3-oxoprop-1-en-1-yl)phenoxy]oxane-2-carboxylic acid	2.22	0.018	↑
	Acetyl-L-tyrosine	1.38	0.041	↑
	N-Carbamylglutamate	4.03	0.018	↑
	Thalictroidine	2.27	0.007	↓
	4-Methylimidazole	2.86	0.002	↓
	Dinorpromazine	1.55	0.004	↓
	()-Octanoylcarnitine	2.21	0.041	↓
	Kievitone	2.42	0.005	↓
	15-hydroxyicosanoic acid	2.21	0.031	↓
	Spirolide E	1.16	0.041	↓
	Ginsenoside Rf	1.28	0.010	↓
	Lauryl diethanolamide	1.19	0.031	↓
	4-Fluoromuconolactone	1.03	0.014	↓
	Lamivudine-monophosphate	1.18	0.031	↓
	Succinylcarnitine	1.19	0.014	↓
	Hydroxyprolyl-Isoleucine	1.23	0.031	↓
	L-Carnitine	2.06	0.014	↓
	Uridine	1.36	0.018	↓
	Isoachifolidiene	1.66	0.041	↓
	LysoPA(0:0/18:2(9Z,12Z))	1.15	0.031	↑
	Cis-5-Tetradecenoylcarnitine	2.27	0.041	↑
	Longicamphenylone	1.04	0.031	↑
	PHENACYLAMINE	1.43	0.014	↑
	()-Octanoylcarnitine	1.95	0.041	↑
**F**	Cortolone-3-glucuronide	1.88	0.041	↑
	17-HYDROXYPROGESTERONE	1.93	0.041	↑
	3-hydroxydecanoyl carnitine	2.01	0.018	↑
	Taurocholic acid	2.86	0.041	↑
	5-(1-hydroxypropan-2-yl)isolongifol-4-ene	1.21	0.024	↑
	2-amino-14,16-dimethyloctadecan-3-ol	3.27	0.001	↑

Spisulosine	3.75	0.001	↑
Tomatidine	2.25	0.005	↑
C14:5n-1,3,5,7,9	1.63	0.010	↑
Dodecanoylcarnitine	2.19	0.024	↑
17-Allylestra-1,3,5(10)-triene-3,17beta-diol	1.85	0.031	↑
Succinoadenosine	1.26	0.041	↑
D-Xylono-1,5-lactone	1.11	0.031	↑
()-Camphoric acid	2.78	0.007	↑
(6E,8R,10Z)-8-hydroxy-3-oxohexadecadienoic acid	2.36	0.003	↑
4,11,13,15-Tetrahydroridentin B	2.25	0.024	↑
3-Hydroxydodecanedioic acid	2.32	0.005	↑
Traumatic Acid	2.23	0.018	↑
Rhamnalpinogenin	3.35	0.010	↑
Tanacetol B	2.14	0.031	↑
2-O-beta-D-Glucopyranuronosyl-D-mannose	2.93	0.041	↑
5-(2-Methylpropyl)tetrahydro-2-oxo-3-furancarboxylic acid	1.70	0.014	↑
Polyethylene, oxidized	2.40	0.007	↑
Jasmonic acid	4.26	0.005	↑
3,4-Methyleneazelaic acid	2.39	0.005	↑
2,2'-(3-methylcyclohexane-1,1-diyl)diacetic acid	2.42	0.002	↑
Aflatoxin GM1	3.04	0.014	↑
20-Oxo-leukotriene E4	4.38	0.014	↑
3-alpha-Androstanediol glucuronide	2.01	0.041	↑
3-Ethenyl-4-hydroxy-2,5-dimethylhex-5-en-2-yl acetate	2.68	0.003	↑
Estrone 3-glucuronide	4.82	0.014	↑
13-Oxo-9,11-tridecadienoic acid	1.75	0.003	↑
Pelargonic acid	3.05	0.041	↑
(1'R)-Nepetalic acid	3.76	0.018	↑
Sebacic acid	1.60	0.024	↑
1,4-Ipomeadiol	2.81	0.014	↑
5-Hexyltetrahydro-2-oxo-3-furancarboxylic acid	1.30	0.041	↑
3-Indolepropionic acid	2.40	0.018	↓
Thalictroidine	2.69	0.010	↓
Trimethylamine N-oxide	2.60	0.018	↓
Guanosine	2.00	0.018	↓
()-Enterolactone	2.35	0.041	↓
postin	2.79	0.031	↓
LysoPC(22:1(13Z))	1.68	0.024	↓
PE-NMe(16:1(9Z)/14:1(9Z))	1.06	0.041	↓

PE(15:0/16:1(9Z))	1.41	0.014	↓
PG(a-13:0/i-12:0)	1.35	0.018	↓
LysoPC(20:0/0:0)	1.64	0.024	↓
Retinol	2.28	0.041	↓
LysoPE(0:0/15:0)	1.86	0.018	↓
1-(2,3-Dihydro-6,7-dimethyl-1H-pyrrolizin-5-yl)-2-hydroxy-1-propanone	3.54	0.010	↓
Isocarbostyril	1.18	0.041	↓
Indole-3-carboxylic acid	2.06	0.041	↓
Oxindole	1.54	0.003	↓
Glutamylmethionine	2.75	0.031	↓
N-ACETYLPROLINE	1.33	0.031	↓
3-Formyl-6-hydroxyindole	1.78	0.031	↓
Glycylproline	2.73	0.005	↓
N(6)-Methyllysine	2.71	0.014	↓
P-Cresol glucuronide	1.41	0.024	↓
2-Phenylethanol glucuronide	2.33	0.014	↓
Mosinone A	1.85	0.041	↓
2-Hydroxyacetaminophen sulfate	3.17	0.018	↓
Indoxylsulfuric acid	1.46	0.010	↓
Isoleucylisoleucine	2.38	0.001	↓
Lacosamide-glucuronide	2.07	0.024	↓
LysoPC(16:0)	1.18	0.014	↓
Hydroxybuprenorphine	1.50	0.031	↓
{[(2E)-3-phenylprop-2-en-1-yl]oxy}sulfonic acid	2.91	0.041	↓
4-ethylphenylsulfate	3.35	0.003	↓
4-Ipomeanol	3.27	0.010	↓
Propylparaben	1.89	0.018	↓
P-Tolyl Sulfate	2.88	0.002	↓
Isoleucyl-Tryptophan	1.96	0.003	↓
O-methoxycatechol-O-sulphate	2.17	0.041	↓
Dopamine quinone	1.84	0.004	↓
Dolichyl phosphate D-mannose	1.42	0.007	↓
2-Methylbutyrylglycine	1.47	0.041	↓
L-Serine	2.23	0.004	↓

P-Value < 0.05means significant differences, ‘ ↑ ’ means increasing, ‘ ↓ ’ means decreasing.
